# First Case of VIM-1-like-Producing *Pseudomonas putida* Bacteremia in an Oncohematological Pediatric Patient in Italy

**DOI:** 10.3390/antibiotics12061033

**Published:** 2023-06-09

**Authors:** Venere Cortazzo, Marilena Agosta, Stefania Gaspari, Gianluca Vrenna, Barbara Lucignano, Manuela Onori, Valentina Di Ruscio, Livia Mancinelli, Danielle Domo, Carlo Federico Perno, Paola Bernaschi

**Affiliations:** 1 Microbiology and Diagnostic Immunology Unit, Department of Diagnostic and Laboratory Medicine, Bambino Gesù Children’s Hospital, IRCCS, 00165 Rome, Italy; venere.cortazzo@opbg.net (V.C.); marilena.agosta@opbg.net (M.A.); carlofederico.perno@opbg.net (C.F.P.); 2 Department of Hematology/Oncology, Cell and Gene Therapy, Bambino Gesù Children’s Hospital, IRCCS, 00165 Rome, Italy; 3 Faculty of Medicine and Surgery, Department of Experimental Medicine, Ph.D. Course in Microbiology, Immunology, Infectious Diseases and Transplants (MIMIT), University of Rome Tor Vergata, Via Montpellier 1, 00133 Rome, Italy

**Keywords:** *Pseudomonas putida*, multidrug-resistant, polymicrobial bacteremia, pediatric patient, metallo-β-lactamases, VIM-1-like

## Abstract

Bacterial infections caused by multidrug-resistant (MDR) Gram-negatives are of great concern worldwide, as they are frequently associated with high mortality and morbidity rates. To date, two cases of VIM-2 metallo-β-lactamase (MBL)-producing *Pseudomonas putida* bacteremia have been ever reported in France and Spain between 2004 and 2010. Here, we present the first case of VIM-1-like-producing *P. putida* isolated in blood culture collected from an oncohematological pediatric patient admitted to Bambino Gesù Children’s Hospital (IRCCS) in Rome, Italy.

## 1. Introduction

Recently, the percentage of multidrug-resistant (MDR) Gram-negatives causing bacteremia has increased, and carbapenemase production is one of the most common mechanisms leading to resistance to β-lactams antibiotics.

According to Ambler classification, metallo-β-lactamases (MBL) are class B carbapenemases with active-site zinc. These enzymes are currently divided into subclasses based on a combination of structural features, zinc affinities for the two binding sites, and hydrolysis characteristics [1].

Genes encoding VIM enzymes (*bla*_VIM_) are located within a variety of integron structures, where they have been incorporated as gene cassettes; when these integrons become associated with plasmids or transposons, transfer between bacteria is readily facilitated [2], even in uncommon pathogens. Among them, *Pseudomonas putida* has been recognized as an opportunistic emerging pathogen, causing various infections in newborns, neutropenic, and cancer patients or in patients with risk factors leading to immunosuppression [3].

*P. putida* is a non-lactose fermenting, oxidase-positive Gram-negative bacillus. It is a member of the fluorescent group of Pseudomonas with variable metabolic features. It is not part of normal human bacterial flora, while it is commonly present in the environment and livestock animals, mostly birds; it is thought to have very low pathogenicity in immunocompetent persons [3,4].

Currently, the emergence of MDR *P. putida* has become a cause of concern. Infections caused by carbapenemase-producing *P. putida* isolates have been reported occasionally in critically ill or immunocompromised patients [5]. Five cases of bacteremia caused by carbapenemase-producing *P. putida*, isolated from blood culture, are described in the literature, i.e., three cases due to KPC-2-producing *P. putida* and two cases due to VIM-2-producing *P. putida*.

In 2008, the first case of infection due to KPC-2-producing *P. putida* was reported in Texas, USA [6]. The second and the third case of KPC-2-producing *P. putida* were both found in Brazil. These were reported in a child with lymphoma [7] and in a 72-year-old man with relapsing chronic lymphocytic leukemia [8] (in 2012 and in 2018, respectively). The cases of VIM-2-producing *P. putida* were reported in 2004 in a 59-year-old immunocompromised French patient and in 2010 in a 71-year-old female Spanish patient with a history of hypertension, insulin-dependent diabetes mellitus, stroke, chronic obstructive pulmonary disease, psychiatric, and lymphoproliferative syndrome [9]. Overall, only one case was revealed to be fatal. The patient died 12 days after her first episode of bacteremia, despite aggressive broad-spectrum antimicrobial therapy [6].

To the best of our knowledge, no bacteremia caused by MBL-producing *P. putida* has been reported in pediatric patients. Here, we report the microbiological and clinical characteristics of a non-fatal case of polymicrobial bacteremia due to VIM-1-like-producing *P. putida* and other Gram-negative bacteria in an oncohematological pediatric patient.

## 2. Case Description

On January 2023, a 7-year-old patient with a diagnosis of acute B cell lymphoblastic leukemia (B ALL) was admitted to the Emergency Department of Bambino Gesù Children’s Hospital for the onset of fever (temperature > 39 °C). The patient received a diagnosis of B ALL in November 2022, and she carried a bilume central venous catheter (CVC). She was given chemotherapy, according to AIEOP BFM ALL 2017 Protocol Consolidation B short, for standard-risk patients.

Blood tests revealed anemia (Hb 9.7 g/dL), neutropenia (total white blood cell of 0.07 × 10^3^ /uL with absolute neutrophil count of 0 × 10^3^ /uL), and thrombocytopenia (PLT 45,000/mmc), with increased indices of inflammation (CRP 2.85 mg/dL, procalcitonin 3 ng/mL). An echocardiogram showed no sign of endocarditis; no parenchymal thickening or pleural effusions have been documented at a chest x-ray.

At admission to the oncology department, the patient presented in fair general conditions with initial signs of dehydration (dehydrated and pale skin, dry tongue, and a slightly elongated capillary refill of 3–4 s). She was febrile. At thorax auscultation, rhythmic and regular cardiac activity and normal air penetration were detected. No abdominal pain was referred, but, at physical examination, an extensive, ulcerated, painful perianal lesion was discovered, with reported perievacuative pain since about two days before. Her physical examination was difficult, given her discomfort. However, the exam demonstrated significant anal and perianal necrosis, granulation tissue without erythema and edema.

At the beginning of the febrile period, blood cultures (BCs) and whole blood samples (for T2 magnetic resonance test, T2MR) were collected from two different central venous catheters (CVC) lines for diagnosis investigation. Moreover, due to the severity of the lesion, perianal swab was collected for microbiological culture. Empirical antibiotic therapies with meropenem, tigecycline, and amikacin were quickly started.

Whole blood samples, processed with a T2Bacteria panel, resulted positive for *Pseudomonas aeruginosa* (in a mean time of 4.4 h). Blood culture tested positive after 17 h of incubation, and Gram-staining revealed the presence of Gram-negative bacilli. BioFire FilmArray blood culture identification 2 (BCID2) panel confirmed positivity for *P. aeruginosa*. The next day, microbiological culture of perianal wound swab was positive for *P. aeruginosa*, too. On the same day, CVC was removed, and two peripheral accesses were placed.

Blood cultures from alternate lumens were repeated at every subsequent peak of fever. Patient received supportive therapies, including intravenous fluids, emocomponent transfusions, and granulocyte colony-stimulating factor (G-CSF).

After the first days of hospitalization, the patient developed respiratory distress with desaturation and reduced respiratory exchanges on blood gas analysis. At examination, a reduction in vesicular murmur on the right side was detected. A CT scan was performed, showing widespread densitometric dishomogeneity of bilateral lung parenchyma due to the presence of multiple and extensive ground-glass areas associated with more consolidative areas with predominantly perihilar-distribution at both upper lobes, with moderate bilateral pleural effusions. A non-invasive respiratory support was needed, and on the second day of recovery, a high-flow nasal cannula (HFNC) was started, with an improvement of oxygen saturation and air penetration in both lungs.

Meantime, the patient continued to be febrile, about every 4–5 h, though with a slow progressive improvement of the thermal curve, achieving a persistent defervescence seven days later.

However, the clinical presentation of perianal lesion did not improve and, after 5 days of hospitalization, blood cultures collected from the peripheral vein resulted in positive result. Gram-staining evidenced again the presence of Gram-negative bacilli and BCID2 panel was performed to detect the aetiological agent and related resistance genes.

This time, beyond the identification of *P. aeruginosa*, BCID2 panel also detected *Enterobacter cloacae complex* target and CTX-M and VIM resistance genes. Species identification of isolated colonies confirmed the BCID2 positivity for *E. cloacae* and *P. aeruginosa* and revealed the presence of two additional off-panel microorganisms, i.e., *Empedobacter brevis* and *Pseudomonas putida*. The immunoassays revealed that *E. cloacae* and *P. putida* were the CTX-M and VIM-producing strains, respectively. Antimicrobial susceptibility testing phenotypically confirmed, as expected, that *E. cloacae* was the CTX-M-producing strain and *P. putida* was the VIM-producing strain, with an associated multidrug-resistant profile.

Finally, based on microbiological results (i.e., the isolation of *E. brevis*, CTX-M-producing *E. cloacae*, *P. aeruginosa*, and VIM-1-like-producing *P. putida*), antibiotic therapy was rapidly modified. According to antimicrobial agent susceptibilities, drugs typically active against these multi-drug resistant pathogens, such as ceftazidime/avibactam and ceftolozane/tazobactam, were administered.

**Figure 1:**
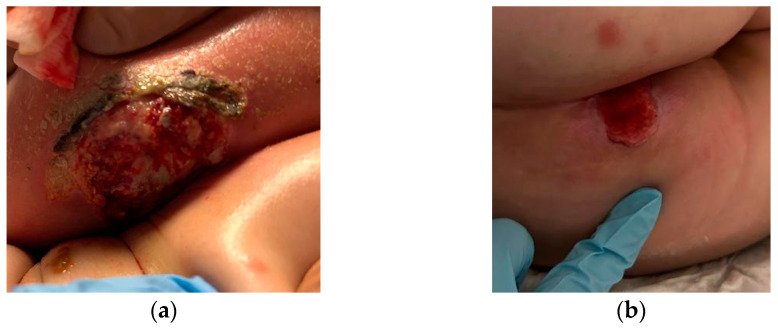
Perianal lesion on day 1 (**a**) and day 50 (**b**) of hospitalization.

After five days of therapy, blood cultures, and perianal swabs were negative. The perianal lesion was treated with local medication with a complete *restitutio ad integrum* after about 50 days (Figure 1). The patient was discharged after 60 days of recovery in good clinical conditions, continuing chemotherapy scheduled for her risk group.

## 3. Microbiological Investigation and Relevant Findings

**Figure 2:**
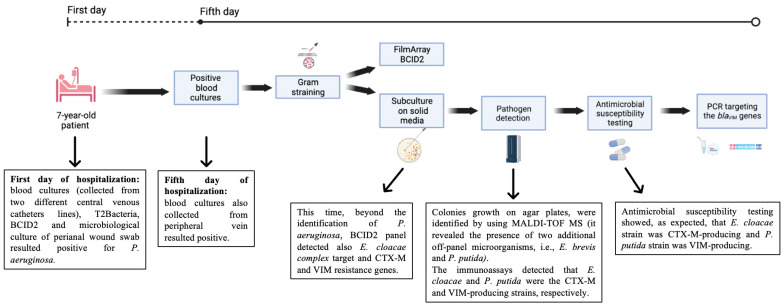
Timeline of microbiological investigations and relevant findings, starting from the fifth day of patient hospitalization (created with BioRender.com, accessed on 29 May 2023. Reproduction of this figure requires permission from Bio.Render.com). Abbreviations: BCID2, BioFire FilmArray blood culture identification 2.

Here, we report the microbiological investigations carried out for diagnosing a polymicrobial bacteriemia caused by *Empedobacter brevis*, CTX-M-producing *Enterobacter cloacae*, *Pseudomonas aeruginosa* and VIM-1-like-producing *Pseudomonas putida* in an oncohematological pediatric patient. Early identification of etiological agents and detection of antimicrobial resistance genes were essential to promptly administer a targeted therapy in such a fragile patient (Figure 2).

T2Bacteria (T2 Biosystems, Lexington MA, USA) was performed for a direct-from-blood rapid identification and multiplex detection of the ESKAPE bacteria (*Enterococcus faecium*, *Staphylococcus aureus*, *Klebsiella pneumoniae*, *Acinetobacter baumannii*, *Pseudomonas aeruginosa*, and *Escherichia coli*) most commonly involved in bloodstream infections [10]. Blood cultures were performed in accordance with hospital practices and international recommendations for pediatric populations [11,12,13]. Each BC bottle was inoculated with 1–3 mL (Bactec Peds Plus/F medium) or 8–10 mL (Bactec Plus Aerobic/F medium, Bactec Lytic/10 Anaerobic/F medium vials, Mycosis IC/F Bactec) of whole blood and incubated at 35 °C in a Bactec 9240/70FX BC system (BD Diagnostics).

According to the internal procedure, 200 µL of positive blood culture were immediately processed with BioFire FilmArray blood culture identification 2 (BCID2) panel (bioMérieux, Marcy l’Etoile, France), for pathogens and resistance genes identification. BCID2 panel enables multiplex detection of 43 molecular targets associated with bloodstream infection, including 15 Gram-negative bacteria, 11 Gram-positive bacteria, 7 Candida species, and 10 antimicrobial resistance genes.

Positive blood cultures were subcultured on solid media, and agar plates were incubated at 37 °C overnight. The following day, colonies grown on agar plates were identified by using Matrix-Assisted Laser Desorption Ionization-Time of Flight Mass Spectrometry (MALDI-TOF MS; Bruker Daltonics, Bremen, Germany).

In addition, the immunochromatographic assays NG-Test CTX-M MULTI and NG-Test Carba 5 (NG Biotech, Guipry, France) were used to rapidly detect (15 min) the five major groups in the CTX-M-type enzymes of extended-spectrum β-lactamases (ESBLs) and the five main carbapenemases (i.e., KPC, OXA-48-like, NDM, VIM, and IMP).

Strains were tested for their antimicrobial susceptibility by VITEK^®^2 (bioMérieux, Marcy-l’Étoile, France) automated system and by broth microdilution method, using the Sensititre Gram Negative DMKGN plate (ThermoFisher Scientific, Waltham, MA, USA), according to clinical breakpoints based on the European Committee on Antimicrobial Susceptibility Testing (EUCAST) tables (version 13.0) [14]. It should be noted that MICs for *E. brevis* were determined, but no clinical interpretation was provided, as clinical breakpoints are not available on EUCAST tables. The MICs obtained for tested strains are presented in Table 1.

Due to the peculiarity of this finding, molecular characterization of *P. putida* VIM resistance gene was performed by Polymerase Chain Reaction (PCR) targeting the *bla*_VIM_ genes. The open reading frame of the *bla*_VIM_ gene was amplified by PCR using two pairs of primer sequences (VIM FW: 5′-GATGGTGTTTGGTCGCATA-3′; VIM RV: 5′- CGAATGCGCAGCACCAG-3′), following the conditions described by Poirel and colleagues. Briefly, amplification was carried out with the following thermal cycling conditions: 10 min at 94 °C and 36 cycles of amplification consisting of 30 s at 94 °C, 40 s at 52 °C, and 50 s at 72 °C, with 5 min at 72 °C for the final extension [15].

**Figure 3:**
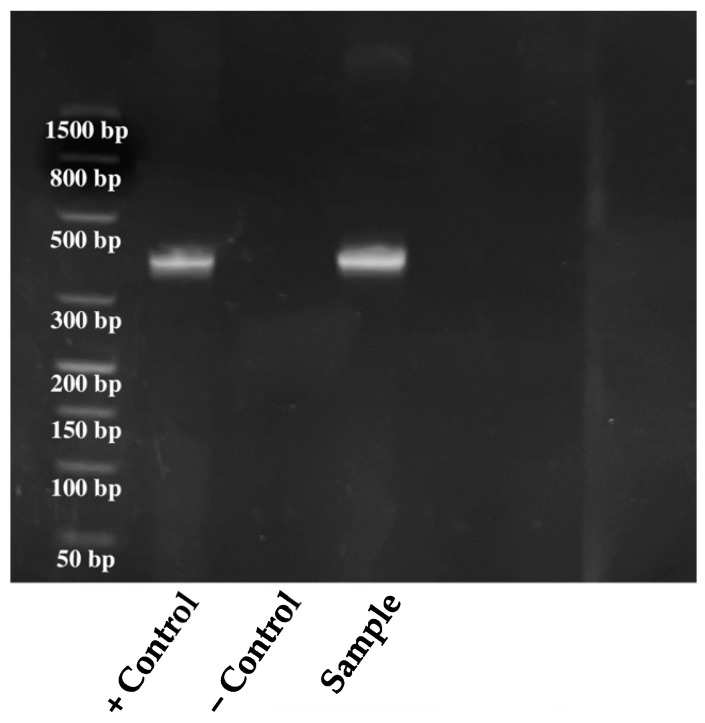
Agarose gel electrophoresis image of the studied sample (*bla*_VIM_ size 390 bp). bp, base pairs; +, positive; −, negative.

Amplification products were resolved and visualized directly on a closed, ready-to-use 2.2% agarose gel-cassette system (FlashGel—Lonza, Switzerland) using the 100 bp FlashGel DNA marker. The fragment obtained was compared to a positive and negative control (Figure 3).

Sequence analysis revealed that the nucleotide sequence was completely homologous to *bla*_VIM-1_-like (GATGGTGTTTGGTCGCATATCGCAACGCAGTCGTTTGATGGCGCGGTCTACCCGTCCAATGGTCTCATTGTCCGTGATGGTGATGAGTTGCTTTTGATTGATACAGCGTGGGGTGCGAAAAACACAGCGGCACTTCTCGCGGAGATTGAAAAGCAAATTGGACTTCCCGTAACGCGTGCAGTCTCCACGCACTTTCATGACGACCGCGTCGGCGGCGTTGATGTCCTTCGGGCGGCTGGGGTGGCAACGTACGCATCACCGTCGACACGCCGGCTAGCCGAGGCAGAGGGGAACGAGATTCCCACGCATTCTCTAGAAGGACTCTCATCGAGCGGGGACGCAGTGCGCTTCGGTCCAGTAGAGCTCTTCTATCCTGGTGCTGCGCATTCG).

In fact, as previously described, sequence alignment of the most common *bla*_VIM_ genes (*bla*_VIM-1_, *bla*_VIM-2_, *bla*_VIM-3_, *bla*_VIM-4_, *bla*_VIM-5_, *bla*_VIM-6_, *bla*_VIM-10_, *bla*_VIM-11_, and *bla*_VIM-12_) revealed that these could be divided into two groups, named *bla*_VIM-1_-like (*bla*_VIM-1_, *bla*_VIM-4_, *bla*_VIM-5_, and *bla*_VIM-12_) and *bla*_VIM-2_-like (*bla*_VIM-2_, *bla*_VIM-3_, *bla*_VIM-6_, *bla*_VIM-10_, and *bla*_VIM-11_) [16]. Moreover, Ellington MJ. and colleagues reported that, based on their DNA and protein sequences, VIM MBLs could be split into three subgroups, i.e., VIM-1-like, VIM-2-like, and VIM-7 [17].

Our findings highlight both the emergence of a frequent resistance mechanism (VIM-1-like) among an uncommon agent of bacteremia and then the potential ability of *P. putida* to acquire resistance genes and become an MDR pathogen.

## 4. Conclusions

The dissemination of *bla*_VIM_ gene in emerging Gram-negative pathogens is of growing concern for clinicians since infections caused by carbapenemase-producing bacteria usually have limited therapeutic options. Reducing the time to microbiological diagnosis of bloodstream infection is important to enable adequate pathogen-based antimicrobial therapy at an early stage and to improve outcomes [18]. In this case, rapid microbiological approaches, i.e., T2MR, FilmArray BCID2, and MALDI-TOF MS, revealed essential for the early detection of pathogens and resistance genes. These tools were useful to guide therapeutic choice and to adopt adequate preventive measures.

*P. putida*, although infrequently isolated from clinical samples, may occasionally cause difficult-to-treat nosocomial infections in severely ill patients. In addition, the presence of an atypical resistant gene in *P. putida* makes even more complex the clinical approach to this patient and to this infection.

We emphasize that, to the best of our knowledge, this is the first case of VIM-1-like-producing *P. putida* polymicrobial bacteremia described worldwide. This underlines the need to monitor the spread of resistance genes also beyond the usual agents of infection.

## Figures and Tables

**Table 1:** Antimicrobial agent susceptibilities of three clinical isolates according to EUCAST interpretative criteria and MIC values for *E. brevis*.

Antimicrobial Agent	Antimicrobial MIC ^1^ (µg/mL) and Interpretation for:							
	*E. cloacae*		*P.aeruginosa*		*P. putida*		*E. brevis*	
	MIC ^1^	INT ^2^	MIC ^1^	INT ^2^	MIC ^1^	INT ^2^	MIC ^1^	INT ^2^
Amikacin	4	S	(4)	-	(4)	-	≤4	-
Amoxicillin/clavulanic	≥32	R	-	-	-	-	8	-
Aztreonam	-	-	4	I	8	I	1	-
Cefepime	≥32	R	-	-	-	-	-	-
Cefotaxime	≥64	R	-	-	-	-	8	-
Ceftazidime	32	R	8	I	>16	R	4	-
Ceftazidime/avibactam	0.5	S	4/4	S	>16/4	R	4	-
Ceftolozane/tazobactam	1	S	2/4	S	>32/4	R	4	-
Ciprofloxacin	≥4	R	0.5	I	0.25	I	≥2	-
Colistin	-	-	2	S	0.5	S	≥8	-
Gentamicin	≥16	R	4	IE	≤0.5	IE	≤0.5	-
Imipenem	≤0.25	S	≤0.5	I	>16	R	1	-
Meropenem	≤0.25	S	0.25	S	>16	R	2	-
Piperacillin/tazobactam	-	-	0.5	I	>32/4	R	2	-
Tobramycin	≥16	R	(≤1)	-	(≤1)	-	≥8	-
Trimethopim/sulfametoxazole	≥320	R	-	-	-	-	≤1	-

^1^ MIC, minimum inhibitory concentration; ^2^ INT, clinical interpretation; S, susceptible; R, resistant; I, susceptible by increased exposure; IE, insufficient evidence that the organism is a good target for therapy with the agent; -, no breakpoints available; ( ), breakpoints in brackets for systemic infections, aminoglycosides should be used in combination with other active therapy.

## Data Availability

All data are described within the text.
